# Supplementary material for: Nicotine engages a VTA-NAc feedback loop to inhibit amygdala-projecting dopamine neurons and induce anxiety-like behaviors
Source: Nat Commun. 2025 Jul 4;16:6196. doi: 10.1038/s41467-025-61180-8 (PMC12227735; doi:10.1038/s41467-025-61180-8)
Supplement: Supplementary file 1 — Supplementary Information [file 41467_2025_61180_MOESM1_ESM.pdf]

**Supplementary information**

# Nicotine engages a VTA-NAc feedback loop to inhibit amygdala-projecting dopamine neurons and induce anxiety-like behaviors.

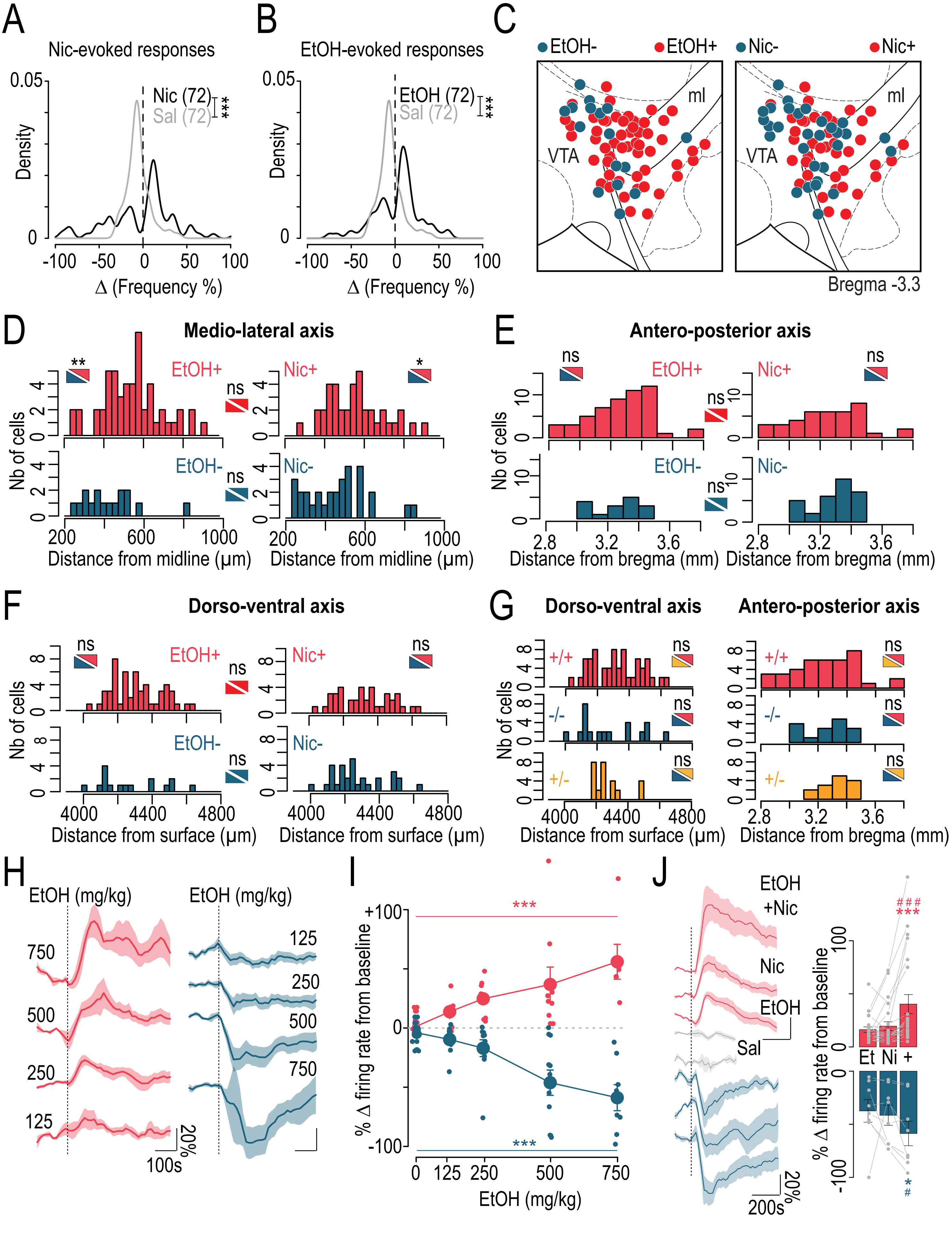

A
Nic-evoked responses
Nic (72)
Sal (72)
***
Density
Δ (Frequency %)
B
EtOH-evoked responses
EtOH (72)
Sal (72)
***
C
EtOH-
EtOH+
Nic-
Nic+
ml
VTA
Bregma -3.3
D
Medio-lateral axis
Nb of cells
Distance from midline (µm)
E
Antero-posterior axis
Distance from bregma (mm)
F
Dorso-ventral axis
Distance from surface (µm)
G
Dorso-ventral axis
Antero-posterior axis
+/+
-/-
+/-
H
EtOH (mg/kg)
100s
20%
I
% Δ firing rate from baseline
EtOH (mg/kg)
J
EtOH +Nic
Nic
EtOH
Sal
200s
Et
Ni
+


Figure S1

**Figure S1:** ***In vivo*** **juxtacellular recordings of VTA DA neurons in response to nicotine and ethanol.** ***Related to Figure 1.***

**(A)** Density of responses evoked by i.v. injection of nicotine (Nic; black, n = 72) or saline (Sal; gray, n = 72) expressed as a percentage of the change in firing frequency induced by the injection (Kolmogorov-Smirnov test, D = 0.44, ***p = $8.9e^{-07}$).
**(B)** same for ethanol (EtOH; black, n = 72 and Sal; gray, n = 72, Kolmogorov-Smirnov test, D = 0.46, ***p = $3.4e^{-07}$).
**(C)** Left: localization of DA neurons activated (EtOH+; red; n = 53) and inhibited (EtOH-; blue; n = 16) by i.v. injection of ethanol, positioned on a Paxinos atlas slice at 3.3 mm from bregma, from neurobiotin-filled cell bodies of all recorded neurons. Right: same for DA neurons activated (Nic+; red; n = 39) and inhibited (Nic-; blue; n = 30) by i.v. injection of nicotine.
**(D)** EtOH- neurons had a more medial distribution within the VTA than EtOH+ neurons (Wilcoxon test, **p = 0.006), Nic- neurons had a more medial distribution within the VTA than Nic+ neurons (Wilcoxon test, *p = 0.017). No difference of distribution was observed between EtOH+ and Nic+ neurons nor EtOH- and Nic- neurons (Wilcoxon test, p = 0.76 and p = 0.42)
**(E)** No anteroposterior differences in their distribution were observed between EtOH+ and EtOH- neuron nor between Nic+ and Nic- neurons (Wilcoxon test, p = 0.76 and p = 0.45). No anteroposterior differences in their distribution were observed between EtOH+ and Nic+ neuron nor between EtOH- and Nic- neurons (Wilcoxon test, p = 0.64 and p = 0.55).
**(F)** No dorsoventral differences in their distribution were observed between EtOH+ and EtOH- neurons nor between Nic+ and Nic- neurons (Wilcoxon test, p = 0.37 and p = 0.26). No dorsoventral differences in their distribution were observed between EtOH+ and Nic+ neuron nor between EtOH- and Nic- neurons (Wilcoxon test, p = 0.76 and p = 0.77).
**(G)** Left: no dorsoventral differences in their distribution were observed between EtOH+/Nic+ (+/+; red; n = 39) and EtOH-/Nic- (-/-; blue; n = 16) neurons (Wilcoxon test, p = 0.31) nor between these neurons and EtOH+/Nic- (+/-; orange; n = 14) neurons (Wilcoxon test, p = 0.43, p = 0.6). Right: no anteroposterior differences in their distribution were observed between these neurons (Wilcoxon test, p = 0.94, p = 0.24, p = 0.28).
**(H)** Time course of mean change in firing frequency (% of baseline) after i.v. injection of different doses of ethanol for activated (EtOH+; red) and inhibited (EtOH-; blue) VTA DA neurons (125, 250, 500 and 750 mg/kg; n = 30/39, 6/10, 9/10, 9/11 and 6/8, respectively for EtOH+ and EtOH- DA neurons).
**(I)** Dose-response curves in EtOH+ (red) and EtOH- (blue) VTA DA neurons. Responses to different doses of ethanol are expressed as percentage of variation from baseline (one-way ANOVA: dose effect $F_{4,55}$ = 10.83, ***p = $1.5e^{-06}$ and $F_{4,73}$ = 20.64, ***p = $2e^{-11}$, respectively for EtOH+ and EtOH- DA neurons).
**(J)** Left: time course of mean change in firing frequency (% of baseline) after i.v. injection of saline (grey), ethanol (500 mg/kg), nicotine (30 µg/kg) and co-injection of nicotine with ethanol in activated (EtOH+/Nic+; red; n = 23) and inhibited (EtOH-/Nic-; blue; red; n = 8) VTA DA neurons. Right: responses to different injections (ethanol, Et; nicotine, Ni; ethanol and nicotine, +) expressed as percentage of variation from baseline. (For EtOH+/Nic+: Et vs Ni, Wilcoxon test, W = 144, p = 0.86 ; Et vs + and Ni vs +, one-sided Wilcoxon test with Holm corrections, W = 26, ***p = $3.4e^{-04}$; W = 9, ###p = $9.3e^{-05}$; for EtOH-/Nic-: Et vs Ni, paired Student's t-test, $t_7$ =0.31, p = 0.76 ; Et vs + and Ni vs +, one-sided paired Student's t-test with Holm corrections, $t_7$ =2.34, *p = 0.03; $t_7$ =2.6, #p = 0.035).
**Data are presented as mean ± SEM. All statistical tests are two-sided if not stated otherwise.**

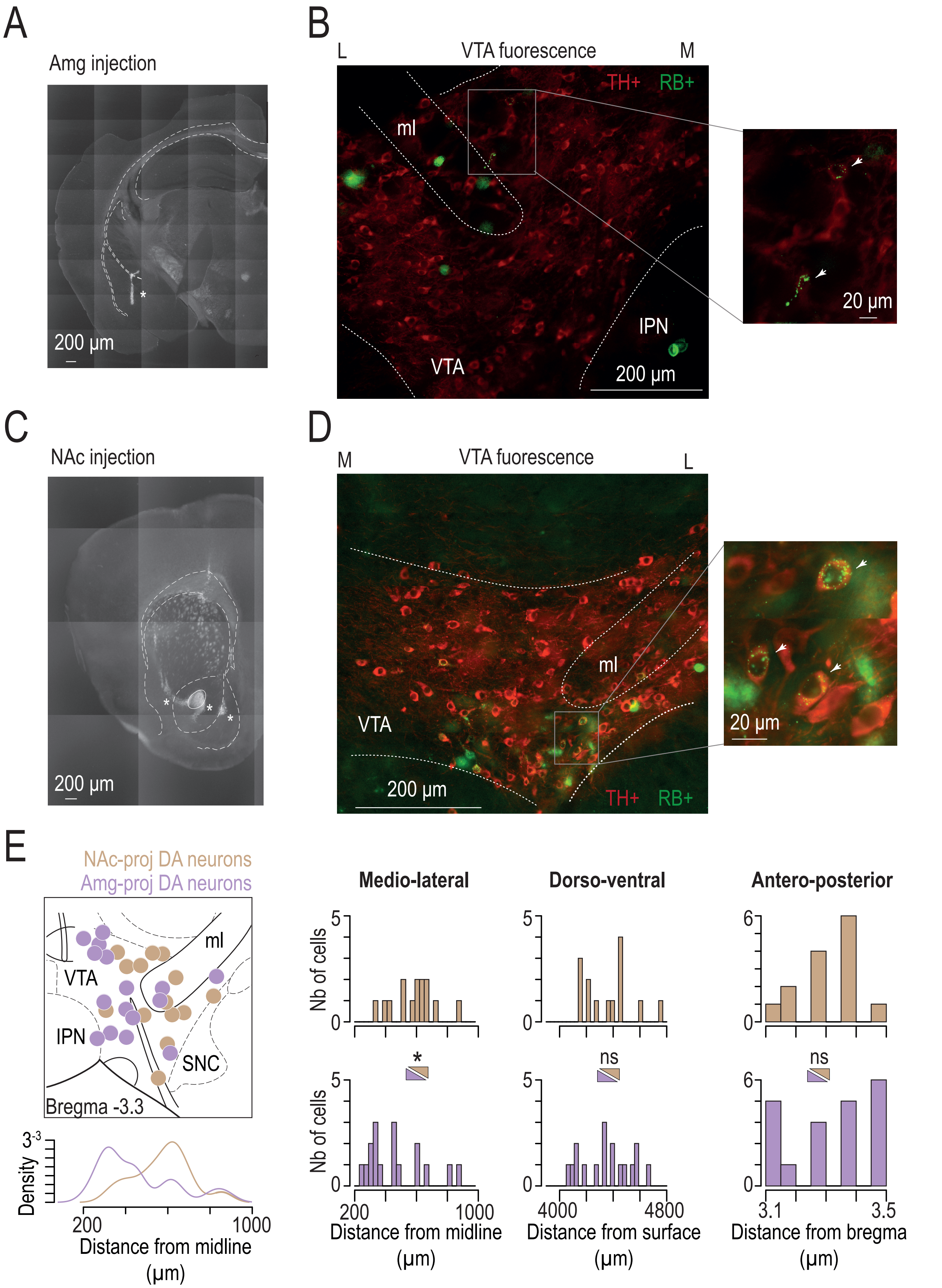
A
Amg injection
200 µm
B
L
VTA fuorescence
M
TH+
RB+
ml
IPN
VTA
200 µm
20 µm
C
NAc injection
200 µm
D
M
VTA fuorescence
L
ml
VTA
200 µm
TH+
RB+
20 µm
E
NAc-proj DA neurons
Amg-proj DA neurons
ml
VTA
IPN
SNC
Bregma -3.3
Density
200
1000
Distance from midline
(µm)
Medio-lateral
Dorso-ventral
Antero-posterior
Nb of cells
*
ns
ns
200
1000
Distance from midline
(µm)
4000
4800
Distance from surface
(µm)
3.1
3.5
Distance from bregma
(µm)

Figure S2

**Figure S2: Identification of NAc- and Amg-projecting DA neurons.** ***Related to Figure 1.***

**(A)** Example of retrobead (RB) injection sites, indicated by asterisks, in the amygdala (Amg), mapped onto a Paxinos atlas slice.
**(B)** Representative immunofluorescence image of VTA slices (TH+, red) revealing neurons containing RB (RB+, green) after RB injection in the Amg.
**(C-D)** Same as (A-B) but for RB injection in the nucleus accumbens (NAc).
**(E)** Localization of Amg-projecting (purple; n = 16) and NAc-projecting (brown; n = 13) VTA DA neurons, positioned on a Paxinos atlas slice at 3.3 mm from bregma, from neurobiotin-filled cell bodies of all recorded neurons. Amg-projecting DA neurons had a more medial distribution within the VTA than NAc-projecting DA neurons (Wilcoxon test, W = 173, *p = 0.03), but neither anteroposterior (Wilcoxon test, W = 111.5, p = 0.77) nor dorsoventral (Welch two sample t-test, $t_{28.159} = 0.26$, p = 0.79) differences in their distribution were observed.
**All statistical tests are two-sided.**

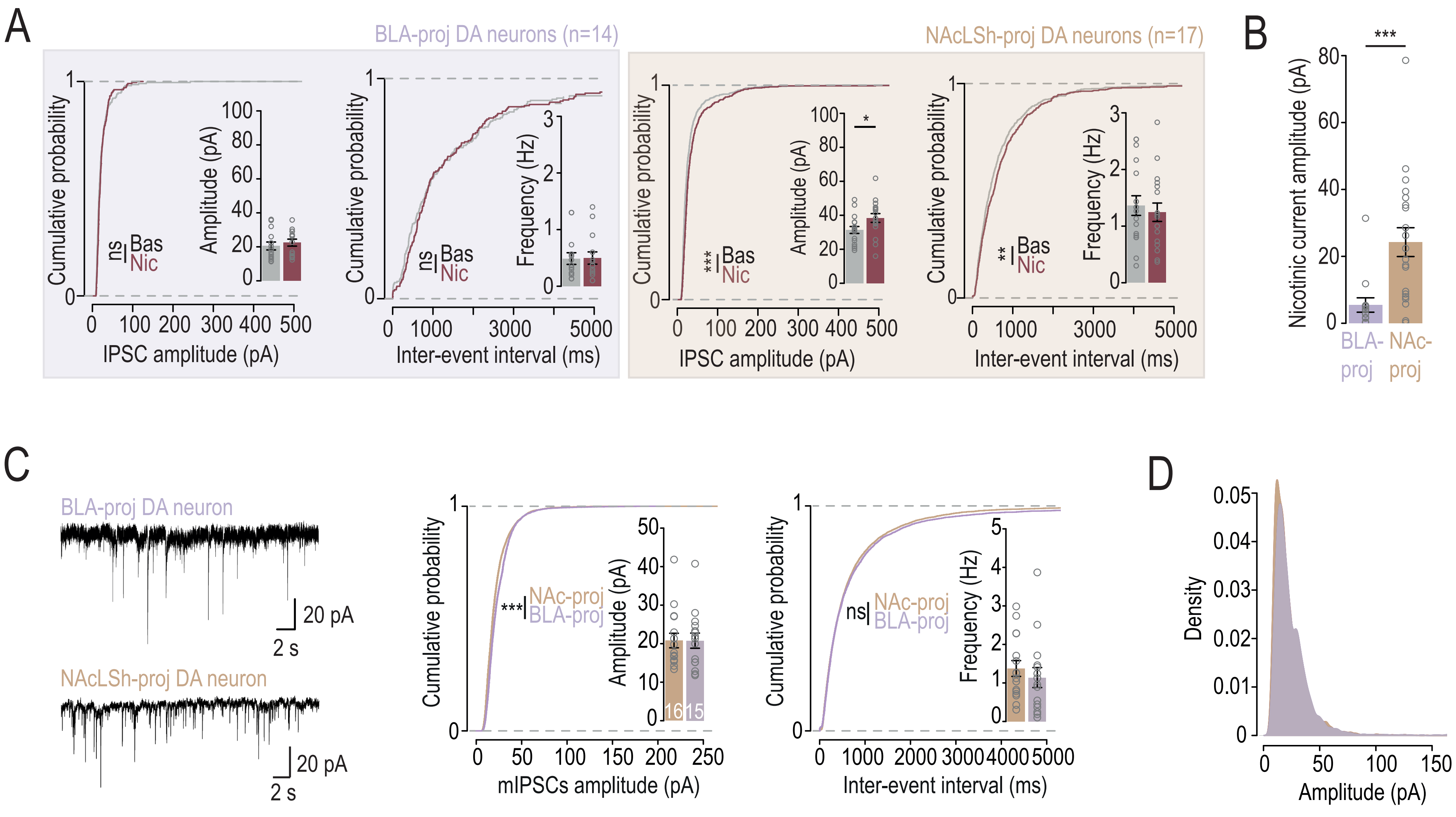
A
BLA-proj DA neurons (n=14)
NAcLSh-proj DA neurons (n=17)
Cumulative probability
IPSC amplitude (pA)
Inter-event interval (ms)
Amplitude (pA)
Frequency (Hz)
Bas
Nic
ns
***
**
*
B
Nicotinic current amplitude (pA)
BLA-proj
NAc-proj
C
BLA-proj DA neuron
NAcLSh-proj DA neuron
20 pA
2 s
mIPSCs amplitude (pA)
NAc-proj
BLA-proj
D
Density
Amplitude (pA)

# Figure S3

**Figure S3: Patch-clamp recordings of spontaneous and miniature inhibitory post-synaptic currents in VTA DA neurons.** ***Related to Figure 2.***

**(A)** Cumulative probability and mean plots of sIPSC amplitudes and frequencies, before (gray) and after (red) bath application of nicotine (30 µM), from VTA DA neurons projecting to the NAcLSh (brown) or to the BLA (purple; Kolmogorov-Smirnov test for distributions; NAc-proj sIPSC amplitude: D=0.16, ***p = $9.1e^{-08}$; NAc-proj sIPSC frequency: D = 0.08, p = 0.02; Welch two sample t-test for mean plots; NAc-proj sIPSC amplitude, $t_{30.343}$ = -2.135, p = 0.04).
**(B)** Mean amplitude of nicotinic inward currents evoked by bath application of nicotine (30 µM), concurrent with IPSCs, in either NAcLSh- (brown; n = 17) or BLA-projecting (purple, n = 14) VTA DA neurons (Wilcoxon test, W = 28, ***p = 0.0001).
**(C)** Example electrophysiological traces of mIPSCs from NAc- or BLA-projecting VTA DA neurons. Cumulative probability and mean plots of mIPSC amplitudes and frequencies from the two populations are represented (Kolmogorov-Smirnov test for amplitudes: D = 0.09, ***p = $2.2e^{-16}$).
**(D)** Density plots of mIPSC amplitudes from DA-NAc or DA-BLA VTA neurons.
**Data are presented as mean ± SEM. All statistical tests are two-sided.**

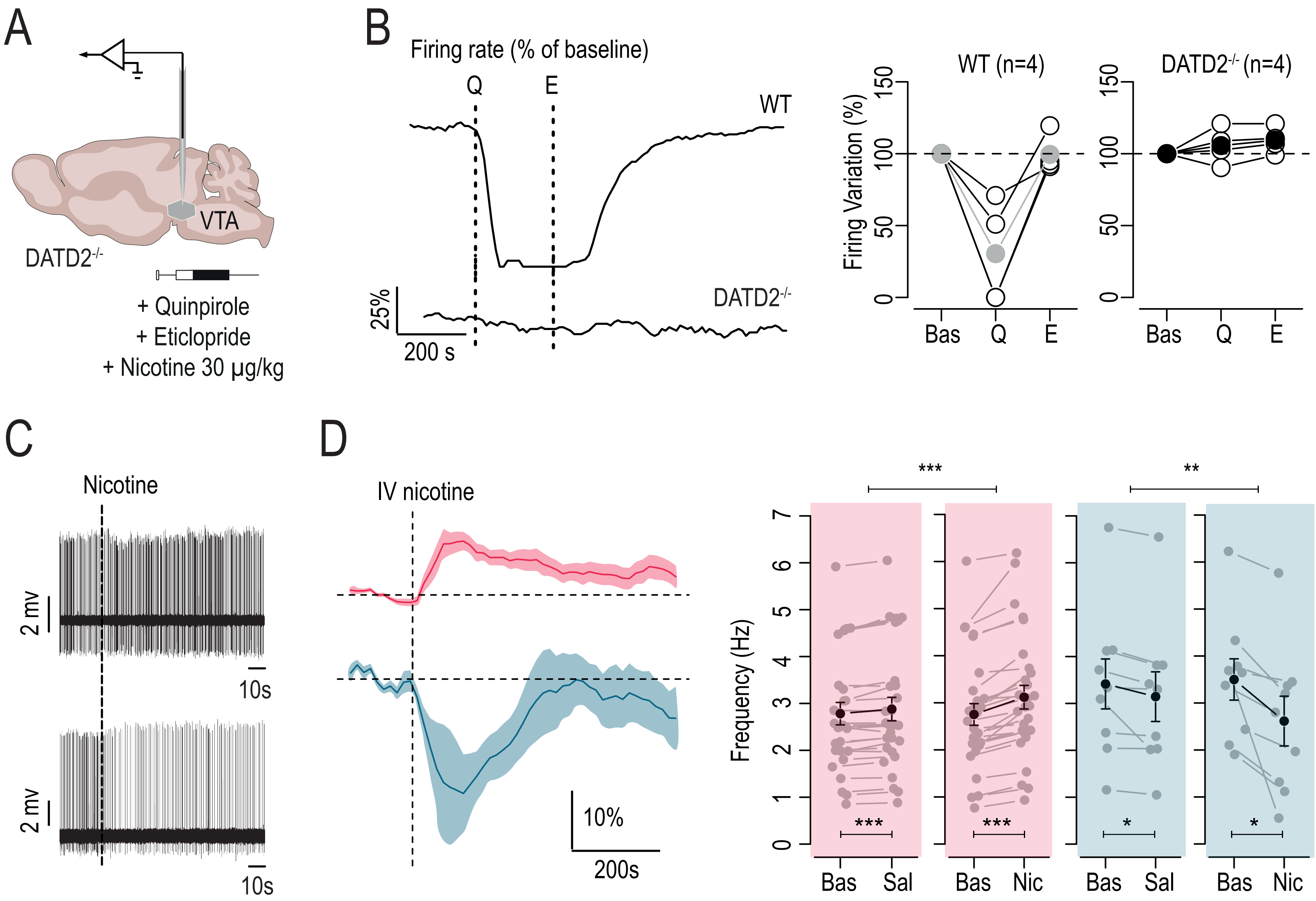
A
DATD2-/-
VTA
+ Quinpirole
+ Eticlopride
+ Nicotine 30 µg/kg
B
Firing rate (% of baseline)
Q
E
WT
DATD2-/-
25%
200 s
WT (n=4)
DATD2-/- (n=4)
Firing Variation (%)
0
50
100
150
Bas
Q
E
C
Nicotine
2 mv
10s
D
IV nicotine
10%
200s
Frequency (Hz)
0
1
2
3
4
5
6
7
***
**
*
Bas
Sal
Nic

# Figure S4

**Figure S4: Nicotine-induced responses in DATD2$^{-/-}$ mice.** ***Related to Figure 2.***

**(A)** *In vivo* juxtacellular recordings were performed on DATD2$^{-/-}$ mice with i.v. injections of quinpirole (1mg/kg), eticlopride (1mg/kg) or nicotine (30 $\mu$g/kg).
**(B)** Example and quantification of change in firing rate variation, expressed as percentage of baseline, after i.v. quinpirole and eticlopride injection, on wild-type (WT; n = 4 ) or DATD2$^{-/-}$ mice (n = 4).
**(C)** Representative recordings of VTA DA neurons activated (*top*) or inhibited (*bottom*) by i.v. injection of nicotine.
**(D)** Left: time course of the mean change in firing frequency, expressed as percentage of baseline, after nicotine i.v. injection in activated (Nic+, n = 28; red) and inhibited (Nic-, n = 9; blue) VTA DA neurons. Right: comparison of firing rate variation (Hz) between baseline (Bas) and saline (Sal) or nicotine (Nic) injection, in Nic+ and Nic- DA neurons. Maximum firing rate after i.v. nicotine for Nic+ neurons or minimum firing rate after i.v. nicotine for Nic- neurons were represented (paired Student's t-test: between Bas vs Sal+, $t_{27} = -4.73$, ***p = 6.2e$^{-05}$, Bas vs Nic+, $t_{27} = -6.82$, ***p = 2.5e$^{-07}$, Bas vs Sal-, $t_8 = 2.5$, *p = 0.04, Bas vs Nic-, $t_8 = 2.93$, *p = 0.02; paired Wilcoxon test: between Bas-Sal vs Bas-Nic, V = 31, ***p = 1.7e$^{-05}$ and V = 43, **p = 0.01 respectively for Nic+ and Nic- neurons).
**Data are presented as mean ± SEM. All statistical tests are two-sided.**

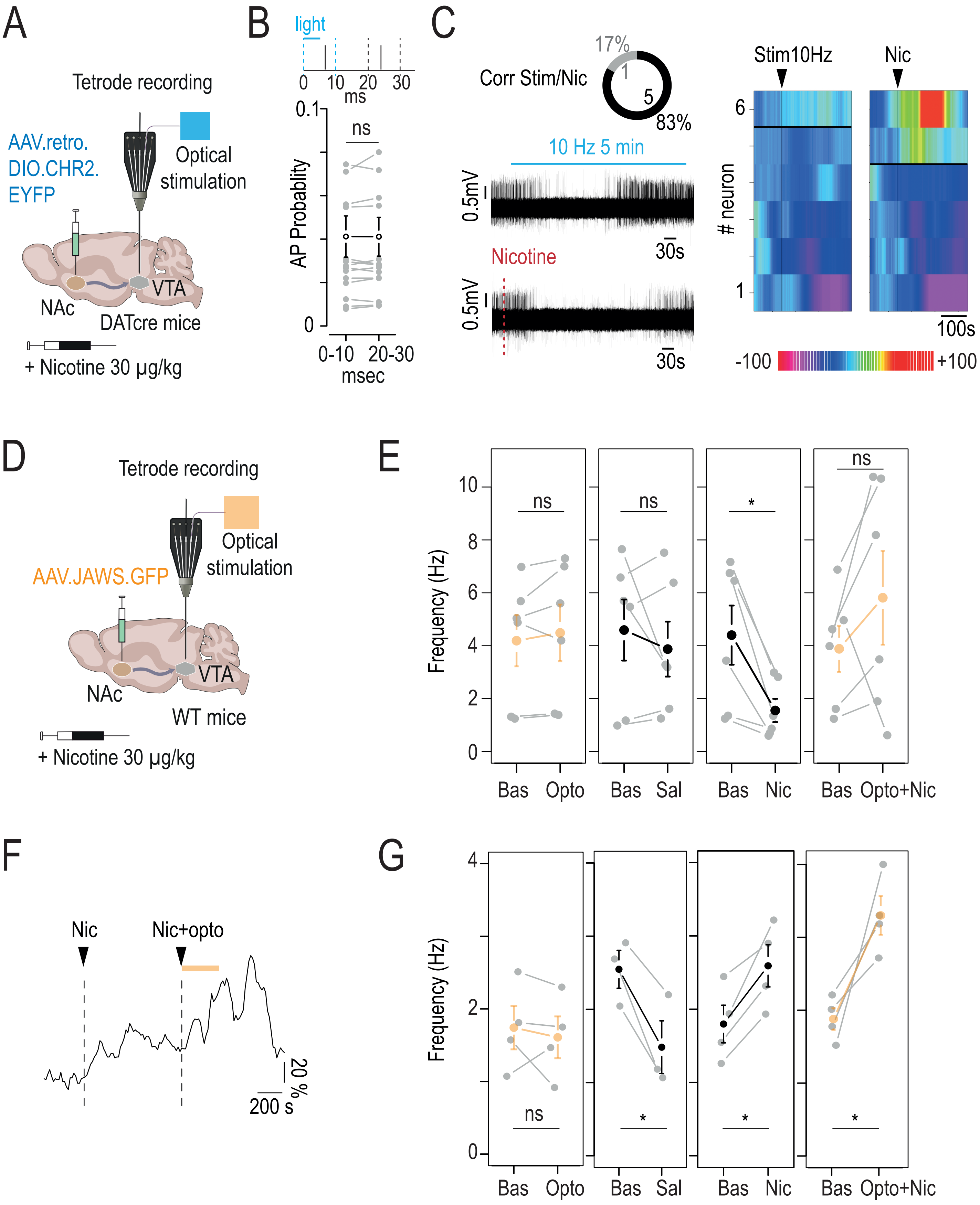
A
Tetrode recording
AAV.retro.
DIO.CHR2.
EYFP
Optical
stimulation
VTA
NAc
DATcre mice
+ Nicotine 30 µg/kg
B
light
ms
ns
AP Probablity
0-10
20-30
msec
C
Corr Stim/Nic
17%
83%
10 Hz 5 min
0.5mV
30s
Nicotine
Stim10Hz
Nic
# neuron
100s
-100
+100
D
Tetrode recording
AAV.JAWS.GFP
Optical
stimulation
VTA
NAc
WT mice
+ Nicotine 30 µg/kg
E
Frequency (Hz)
ns
*
Bas
Opto
Sal
Nic
Opto+Nic
F
Nic
Nic+opto
200 s
20 %
G
Frequency (Hz)
ns
*
Bas
Opto
Sal
Nic
Opto+Nic

Figure S5

**Figure S5: Multiunit recordings of VTA pDA neurons under nicotine and optogenetic modulation of NAc-VTA projections.** ***Related to Figure 3 and 4.***

**(A)** AAV for Cre-dependent expression of retroChR2 was injected in the NAc (NAcLShell + NAcMShell + NAcCore) of DAT-Cre mice, and multi-unit extracellular recordings coupled with i.v. injection of nicotine were performed.
**(B)** Probability of AP occurrence within 10 ms windows following the onset of a 5 ms light pulse or 20 ms after the light pulse was measured in light-activated pDA neurons (n = 15, one data point outside the scale) during a 10 Hz regular stimulation pattern. No difference in AP occurrence was observed during or between light pulses, indicating the absence of phototagging (paired Wilcoxon test, W = 0.78, p = 0.56).
**(C)** Left: representative recordings of VTA DA neurons inhibited by 10 Hz optogenetic VTA-NAc activation (ultra-high-power LED: 600-630 nm; 5 min stimulation; *top*) or by i.v. injection of nicotine (*bottom*). Right: responses of VTA DA neurons to 10 Hz stimulation or i.v. nicotine injection. Responses are ranked based on 10 Hz photostimulation and nicotine response, from most activated (red) to most inhibited (pink; n = 6 neurons). The horizontal line demarcates neurons showing inhibition (5 out of 6 for light and 4 out of 6 for nicotine) from those showing activation in response to light or nicotine.
**(D)** JAWS-expressing AAV was injected in the NAc (NAcLShell + NAcMShell + NAcCore) of WT mice, and multi-unit extracellular recordings coupled with i.v. injection of nicotine were performed.
**(E)** Comparison of firing rate variation (Hz) between baseline (Bas) and 5 minutes of continuous optogenetic inhibition (Opto) or saline (Sal) or nicotine (Nic) or optogenetic inhibition coupled with nicotine injection (Opto+Nic) for nicotine-inhibited putative DA neurons (n = 6; paired Student's t-test: Bas vs Opto, $t_5$ = -1.12, p = 0.32; Bas vs Sal, $t_5$ = 0.8, p = 0.46; Bas vs Nic, $t_5$ = 3.55, *p = 0.02; Bas vs Opto+Nic, $t_5$ = -1.3, p = 0.25).
**(F)** Example of change in firing rate variation expressed as percentage of baseline after i.v. nicotine injection (Nic) or i.v. nicotine injection coupled with optogenetic inhibition of NAc terminals in the VTA (Opto+Nic).
**(G)** Comparison of firing rate variation (Hz) between baseline (Bas) and 5 minutes of continuous optogenetic inhibition (Opto) or saline (Sal) or nicotine (Nic) or optogenetic inhibition coupled with nicotine injection (Opto+Nic) for nicotine-activated putative DA neurons (n = 4; paired Student's t-test: Bas vs Opto, $t_3$ = 0.62, p = 0.58; Bas vs Sal, $t_2$ = 4.56, *p = 0.04; Bas vs Nic, $t_3$ = -4.55, *p = 0.02; Bas vs Opto+Nic, $t_3$ = -3.62, *p = 0.04).
**Data are presented as mean ± SEM. All statistical tests are two-sided.**

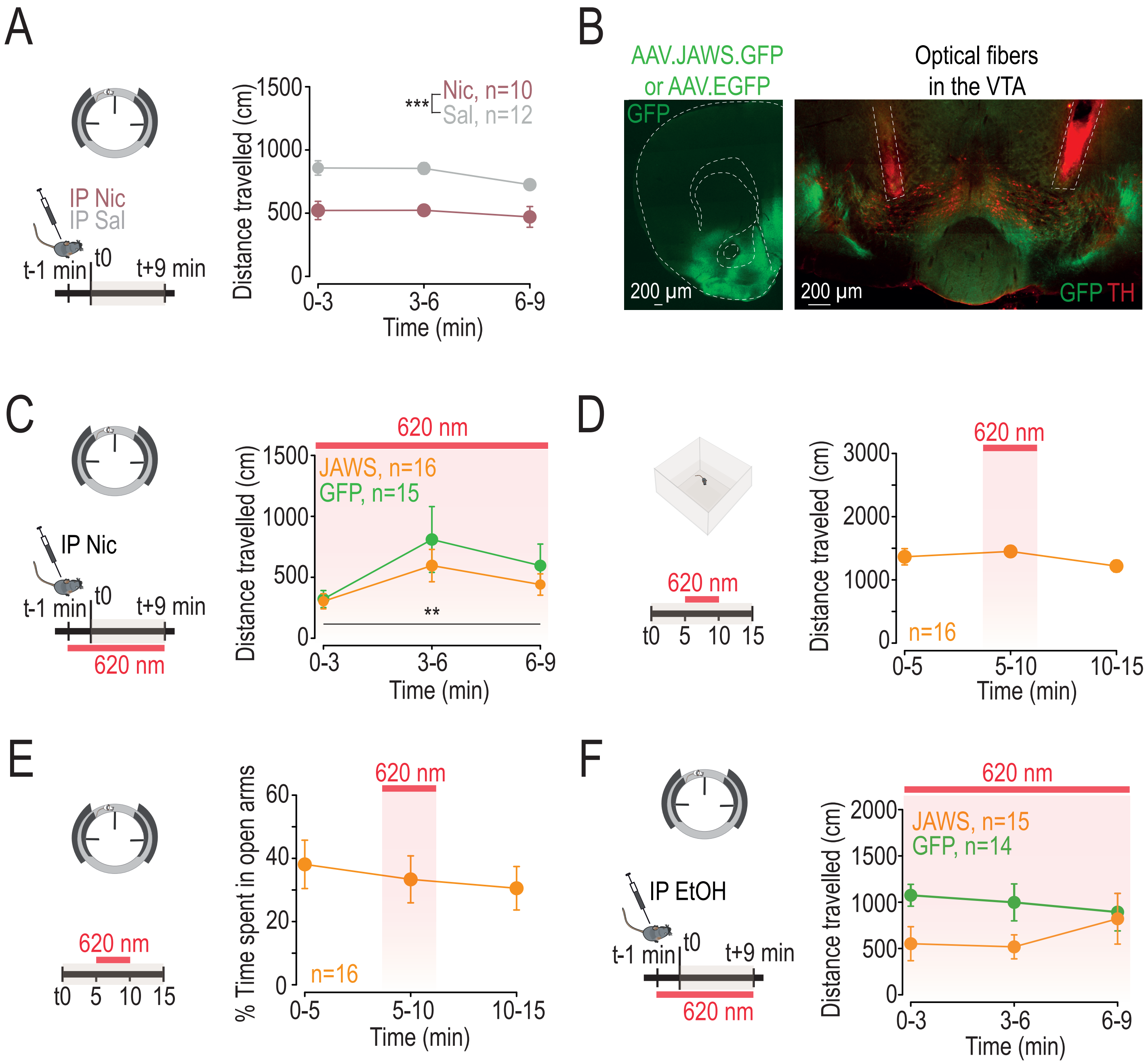
A
IP Nic
IP Sal
t-1 min
t0
t+9 min
Distance travelled (cm)
Nic, n=10
Sal, n=12
***
Time (min)
B
AAV.JAWS.GFP
or AAV.EGFP
GFP
200 µm
Optical fibers
in the VTA
GFP TH
C
IP Nic
620 nm
JAWS, n=16
GFP, n=15
**
D
620 nm
t0 5 10 15
Distance traveled (cm)
n=16
E
% Time spent in open arms
F
IP EtOH
JAWS, n=15
GFP, n=14

Figure S6

**Figure S6: Optogenetic inhibition of NAc terminals in the VTA does not impair locomotion or anxiety-like behavior itself but prevents ethanol-induced anxiety.** ***Related to Figure 4.***

**(A)** Locomotor activity in the elevated O maze (EOM) test. Left: intraperitoneal (i.p.) injection of nicotine (0.5 mg/kg) or saline 1 min before the elevated O maze (EOM) test. Right: distance travelled during the test for saline and nicotine injected mice (n = 10 and 12 respectively; two-way RM ANOVA: group effect, $F_{1,20} = 34.31$, ***p = 9.9e$^{-06}$).
**(B)** JAWS-expressing AAV was injected in the NAc (NAcLSh, NAcMSh, and NAcCore) and optical fibers were implanted in the VTA. Immunohistochemistry shows viral expression in the NAc (GFP; *left*) and of NAc terminals in the VTA (GFP, TH; *right*). Dashed lines indicate the location of the fibers implanted in the VTA.
**(C)** Locomotor activity in the elevated O maze (EOM) test. Left: i.p. nicotine (0.5 mg/kg) was performed 1 min before the EOM test and light stimulation was then continuously maintained throughout the entire test. Right: distance travelled by JAWS- and GFP-expressing mice during the test with continuous inhibition of NAc terminals in the VTA (n = 16 and 15 respectively; two-way RM ANOVA: time effect, $F_{2,58} = 5.9$, **p = 0.005).
**(D)** Locomotor activity was assessed in an open field apparatus. Left: the test lasted 15 minutes and consisted of 5-minute light period (continuous at 620 nm) in between two non-light periods (OFF-ON-OFF). Right: distance travelled of JAWS-expressing mice during the test. No change in locomotor activity was observed upon optogenetic inhibition of NAc terminals in the VTA (n = 16; one-way ANOVA: $F_{1,46} = 0.97$, p = 0.33).
**(E)** Anxiety-like behavior was assessed in the elevated O maze (EOM) test. Left: the test lasted 15 minutes and consisted of 5-minute light period (continuous at 620 nm) in between two non-light periods (OFF-ON-OFF). Right: percentage of time spent in open arms of JAWS-expressing mice during the test. No change in anxiety-like behavior was observed upon optogenetic inhibition of NAc terminals in the VTA (n = 16; one-way ANOVA: $F_{1,46}=0.54$, p=0.46).
**(F)** Locomotor activity in the elevated O maze (EOM) test. Left: i.p. nicotine (1 g/kg) was performed 1 min before the EOM test and light stimulation was then continuously maintained throughout the entire test. Right: distance travelled by JAWS- and GFP-expressing mice during the test with continuous inhibition of NAc terminals in the VTA (n = 15 and 14 respectively; two-way RM ANOVA: no effect of group, $F_{1,27} = 2.95$, p = 0.097; no effect of time, $F_{2,54} = 0.24$, p = 0.78).
**Data are presented as mean ± SEM. All statistical tests are two-sided.**

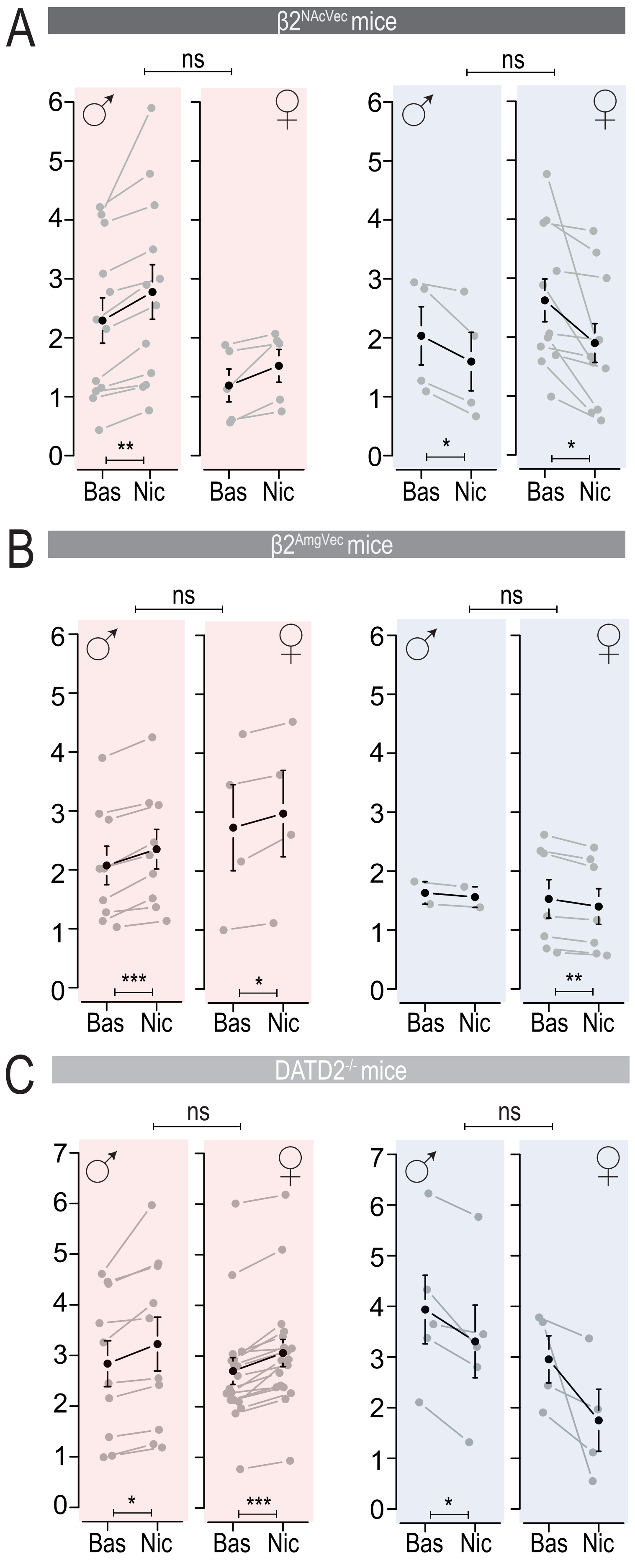


Figure S7

**Figure S7: Comparison of nicotine-induced responses *in vivo* between males and females. *Related to methods.***

**(A)** Comparison of firing rate variation (Hz) between baseline and nicotine injection and between sexes in $\beta 2^{NAcVec}$ mice. Maximum firing rate for activated neurons (red) or minimum firing rate for inhibited neurons (blue) after i.v. nicotine (Paired t-test for Bas vs Nic: activated neurons in males: $t_{11}$ = -3.7, **p=0.003 or in females $t_4$ = -2.56, p = 0.06; inhibited neurons in males: $t_3$ = 3.29, *p = 0.046 or in females $t_{10}$ = 2.89, *p = 0.02; Wilcoxon test for comparison between males and females: for activated neurons, W = 39, p = 0.38 or for inhibited neurons, W = 23, p = 0.95).

**(B)** Same as (A) in $\beta 2^{AmgVec}$ mice (Paired t-test for Bas vs Nic: activated neurons in males: $t_8$ = -5.99, ***p=0.0003 or in females $t_3$ = -3.21, *p = 0.049; inhibited neurons in females $t_6$ = 4.75, **p = 0.003; Welch two sample t-test or Wilcoxon test for comparison between males and females: for activated neurons, $t_{5.44}$ = 0.43, p = 0.69 or for inhibited neurons, W = 10, p = 0.5).

**(C)** Same as (A) in $DATD2^{-/-}$ mice (Paired t-test or Wilcoxon test for Bas vs Nic: activated neurons in males: $t_9$ = -3.09, *p = 0.01 or in females V = 0, ***p = 0.0002; inhibited neurons in males $t_4$ = 3.6, *p = 0.02; Wilcoxon test for comparison between males and females: for activated neurons, W = 76, p = 0.52 or for inhibited neurons, W = 9, p = 0.9).

**Data are presented as mean ± SEM. All statistical tests are two-sided.**
